# Estimating molecular preservation of the intestinal microbiome via metagenomic analyses of latrine sediments from two medieval cities

**DOI:** 10.1098/rstb.2019.0576

**Published:** 2020-10-05

**Authors:** Susanna Sabin, Hui-Yuan Yeh, Aleks Pluskowski, Christa Clamer, Piers D. Mitchell, Kirsten I. Bos

**Affiliations:** 1 Max Planck Institute for the Science of Human History, Jena, Germany; 2 Center for Evolution and Medicine, Arizona State University, Tempe, AZ, USA; 3 School of Humanities, Nanyang Technological University, 48 Nanyang Avenue, Singapore 639818, Singapore; 4 Department of Archaeology, University of Reading, Whiteknights, Reading RG6 6AB, UK; 5 École Biblique de Jérusalem, PO Box 19053, IL9119001, Jerusalem; 6 Department of Archaeology, University of Cambridge, The Henry Wellcome Building, Fitzwilliam Street, Cambridge CB2 1QH, UK

**Keywords:** metagenomics, parasitology, archaeology, aDNA, microbiome

## Abstract

Ancient latrine sediments, which contain the concentrated collective biological waste of past whole human communities, have the potential to be excellent proxies for human gastrointestinal health on the population level. A rich body of literature explores their use to detect the presence of gut-associated eukaryotic parasites through microscopy, immunoassays and genetics. Despite this interest, a lack of studies have explored the whole genetic content of ancient latrine sediments through consideration not only of gut-associated parasites, but also of core community gut microbiome signals that remain from the group that used the latrine. Here, we present a metagenomic analysis of bulk sediment from medieval latrines in Riga (Latvia) and Jerusalem. Our analyses reveal survival of microbial DNA representative of intestinal flora as well as numerous parasites. These data are compared against parasite taxon identifications obtained via microscopy and ELISA techniques. Together, these findings provide a first glimpse into the rich prokaryotic and eukaryotic intestinal flora of pre-industrial agricultural populations, which may give a better context for interpreting the health of modern microbiomes.

This article is part of the theme issue ‘Insights into health and disease from ancient biomolecules’.

## 1. Introduction

Studies of ancient parasites and ancient human gut microbiota in palaeofaeces and latrine sediments have provided a window into past population health, dietary practices and movement. Recent studies have identified eukaryotic parasites through microscopy [1–6], immunoassays [5–9] and genetics [10–20]. These studies have demonstrated the antiquity of many eukaryotic parasites in human populations and have led to proposals of coevolutionary relationships in many cases [21–26]. Investigations of the gut microbiome in palaeofaeces are limited to genetic approaches, and comparatively few studies have been published on this subject [27–30]. By contrast, genetic investigation of latrine sediments has the potential to permit evaluations of whole community gut microbial profiles for both commensal organisms and pathogenic eukaryotic parasites in tandem.

**Figure 1.**
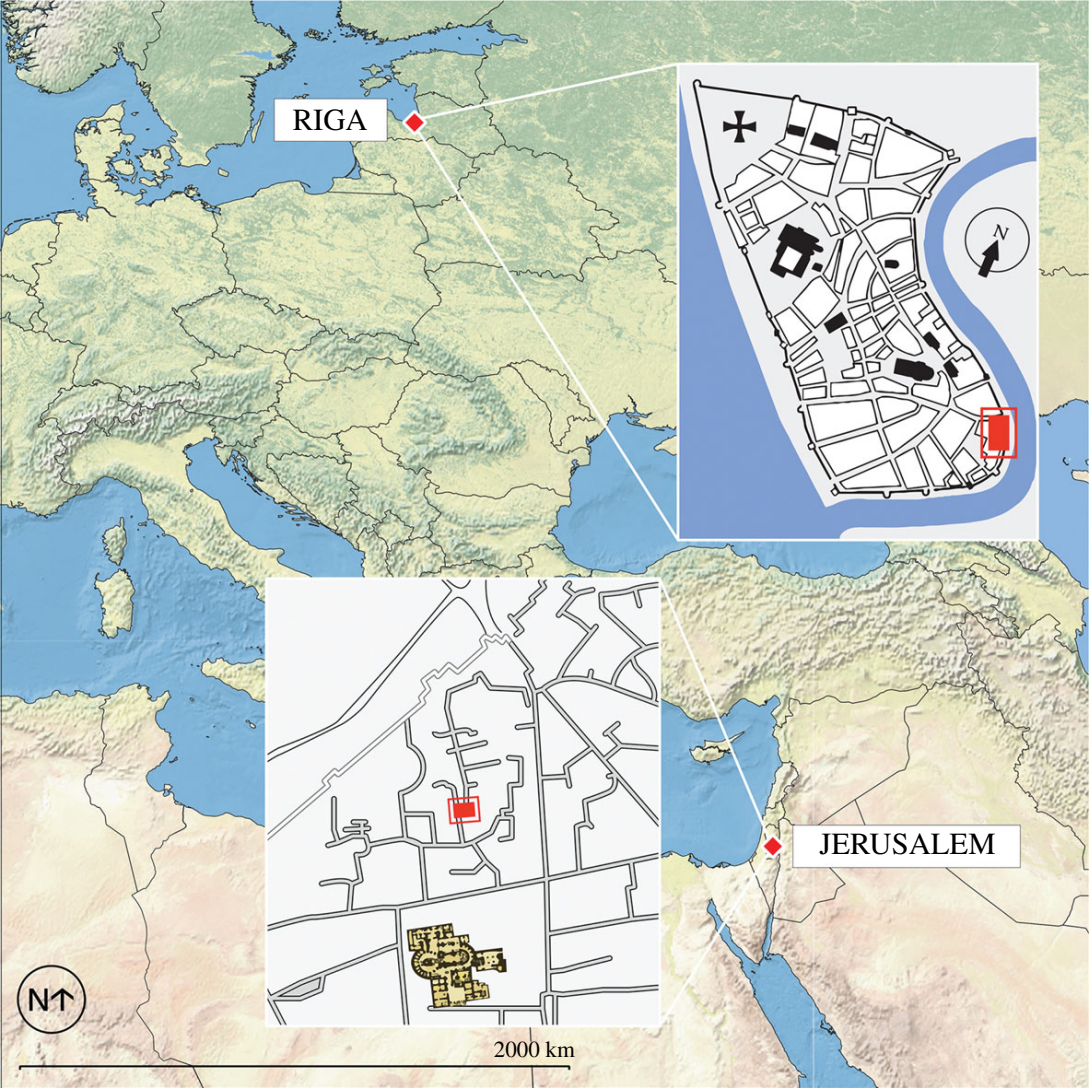
Map indicating locations of the two archaeological sites considered in this analysis. Precise locations of the latrines are shown with bordered red rectangles.

Here, we incorporate ancient DNA (aDNA) with previously published palaeoparasitology investigations at the University of Cambridge Ancient Parasites Laboratory to elucidate the human gastrointestinal microbiome at two archaeological sites, in Jerusalem and Riga, Latvia (figure 1). These two sites were chosen as they are of very similar dates (fourteenth and fifteenth centuries AD) but are located in different regions of the medieval world, and in cities of different antiquity. Sediments from the latrine pits at each site have previously been analysed for helminth eggs using digital light microscopy, and for specific species of protozoan eukaryotic parasites using enzyme linked immunosorbent assays (ELISA) [5,6]. Samples from these sites were provided for metagenomic screening to explore their genetic composition in order to determine the diversity of prokaryotes and eukaryotes, and to compare this approach with the analytical resolution offered by the previous microscopy and ELISA analyses. We also report on possible library inhibition and the results of subsequent template reduction, α-diversity of the total metagenome from each site, estimated source contributions, the presence of specific gut-associated taxa and the preservation of eukaryotic parasite DNA. Following the latter, we discuss similarities and differences between the findings from the original palaeoparasitological analyses and those from the genetic analysis, including the benefits and limitations of the genetic methodology.

### (a) Latrine sediment samples

The Jerusalem latrine was located in the Christian Quarter of the Old City. The city had been continuously inhabited for several thousand years. Chronological estimates obtained through a combination of pottery analysis and radiocarbon dating suggests the latrine was used during the fifteenth century [5]. The cesspit was fed by two separate chutes from separate latrines, suggesting multiple different users from more than one household. Microscopic analyses of latrine sediment and 12 individual coprolites revealed eggs from the helminths *Trichuris trichiura* (whipworm), *Ascaris lumbricoides* (roundworm)*, Taenia* sp. (tapeworm) and *Dibothriocephalus* sp. (fish tapeworm). The protozoans *Entamoeba histolytica* and *Giardia duodenalis* were identified by ELISA [5].

The city of Riga was founded in the twelfth century. The latrine discussed here was located in the Liv Quarter of the city. The wooden structure of the latrine was dated to 1356 CE by dendrochronology [6]. The latrine was located close to a public street, and appears to have been for the general use of the town population. Eggs belonging to the helminths *T. trichiura*, *Dibothriocephalus* sp., *A. lumbricoides* and *Oxyuris equi* (equid pinworm) were identified via microscopy in the Riga latrine sediment, and the protozoan *E. histolytica* was identified by ELISA analysis [6] (table 1).

**Table 1.** Eukaryotic parasites previously identified at the Jerusalem and Riga latrine sites through non-DNA methods.

taxon	description	Jerusalem	Riga
*Ascaris lumbricoides*	causative agent of ascariasis; roundworm; occupies small intestine in host	microscopy	microscopy
*Dibothriocephalus* sp.	causative agent of dibothriocephaliasis; fish tapeworm; occupies small intestine	microscopy	microscopy
*Entamoeba histolytica*	causative agent of invasive amebiasis; protozoan parasite; occupies lumen of colon or cecum, may infect other tissues	ELISA	ELISA
*Giardia duodenalis* (aka *Giardia intestinalis*)	causative agent of giardiasis; protozoan parasite; occupies small intestine in host	ELISA	NA
*Oxyuris equi*	horse pinworm; occupies large intestine in horses	NA	microscopy
*Taenia* sp.	causative agents of taeniasis; beef (*T. saginata*), pork (*T. solium*) or Asiatic (*T. asiatica*) tapeworm; occupy small intestine in host	microscopy	NA
*Trichuris trichiura*	whipworm; can occupy large intestine, caecum and appendix in host	microscopy	microscopy

## 2. Results

### (a) Library inhibition

DNA was extracted from two subsamples of sediment each from the Jerusalem and Riga latrine sites obtained from the same bulk samples that underwent microscopy analysis for parasites. The eluate of the Jerusalem extracts was observed to have a brown tinge. As such discolorations can be indicative of enzymatic inhibition [31], we opted to build one set of Illumina sequencing libraries for Jerusalem using 10 µl of DNA extract (standard for our laboratory) and one set using 2 µl of DNA extract (see Methods). For extracts absent of inhibitory chemicals that could impair enzymatic function, we would expect the libraries built with 10 µl of extract to contain approximately five times as many molecules as the libraries built from 2 µl of extract, as measured by qPCR after ligation and fill-in of Illumina adapters, but prior to indexing and amplification. However, we found that all Jerusalem libraries, regardless of DNA input volume considered here, had approximately the same number of DNA fragments (table 2). This indeed suggested an influence of enzymatic inhibition on detectable DNA quantity, at least for the high-template (10 µl) library. Five additional low-template volume libraries were created for both Jerusalem extracts and carried forward to approximate the complexity that would come from a 10 µl template library with reduced inhibition. All low-template libraries yielded consistent levels of quantifiable DNA. These libraries were subsequently combined for post-indexing processing steps, and formed the basis of all downstream analyses.

**Table 2.** qPCR fragment numbers for pre-indexed libraries. The libraries built from 2 µl of extract have as many or more DNA fragments as measured via qPCR after the ligation of Illumina adapters, but prior to indexing and amplification.

	extract	library ID	library type	mean fragments per µl	standard/reduced fragment number proportion	expected standard/reduced fragment number proportion
Jerusalem	A	1	10 µl template (standard)	1.24 × 10^7^	1.00	1
		2	2 µl template	1.84 × 10^7^	1.49	0.2
		3.1	2 µl template^a^	1.99 × 10^7^	1.61	0.2
		3.2	2 µl template^a^	1.37 × 10^7^	1.11	0.2
		3.3	2 µl template^a^	2.03 × 10^7^	1.64	0.2
		3.4	2 µl template^a^	1.86 × 10^7^	1.51	0.2
		3.5	2 µl template^a^	2.19 × 10^7^	1.77	0.2
	B	1	10 µl template (standard)	3.32 × 10^7^	1.00	1
		2	2 µl template	4.62 × 10^7^	1.39	0.2
		3.1	2 µl template^a^	3.08 × 10^7^	0.93	0.2
		3.2	2 µl template^a^	3.20 × 10^7^	0.97	0.2
		3.3	2 µl template^a^	3.35 × 10^7^	1.01	0.2
		3.4	2 µl template^a^	2.35 × 10^7^	0.71	0.2
		3.5	2 µl template^a^	2.89 × 10^7^	0.87	0.2
Riga	A	1	10 µl template (standard)	2.82 × 10^8^	1.00	1
		2	2 µl template	4.95 × 10^8^	1.76	0.2
	B	1	10 µl template (standard)	2.84 × 10^8^	1.00	1
		2	2 µl template	3.74 × 10^8^	1.32	0.2

^a^The Jerusalem A3.1–5 and B3.1–5 libraries were constructed with 2 µl of template per library, then combined after the indexing step.

Despite the reduced discoloration of the Riga libraries, two low-template libraries were produced from these extracts for comparison. As with Jerusalem, both Riga low-template libraries were indeed richer in quantifiable DNA than the corresponding 10 µl template libraries, thus suggesting the action of enzymatic inhibition here as well (table 2).

### (b) α-Diversity

Species-level α-diversity was evaluated for each library pool with species richness (count of species) and Simpson's diversity index (see Methods). Simpson's diversity index was selected over Shannon's diversity index due to the assumption by Simpon's index of sampling from a population of unknown size and its valuing of more common/abundant species over uncommon/rare species. We assessed these metrics for each individually sequenced library pool, and for computationally combined libraries representative of each site. For the *in silico* combined libraries, we calculated diversity including and excluding human reads (see Methods; electronic supplementary material, figure S1). We used the taxonomic binning tool MALT [32], as implemented in the HOPS pipeline [33], to generate metagenomic profiles for each library. MALT output was visually assessed with MEGAN6 [34], which was then used to produce taxon tables with species-level counts of assigned reads (normalized across the Jerusalem and Riga combined libraries) for all taxonomic groups combined—Bacteria, Archaea, Eukaryota and Viruses.

**Figure 2.**
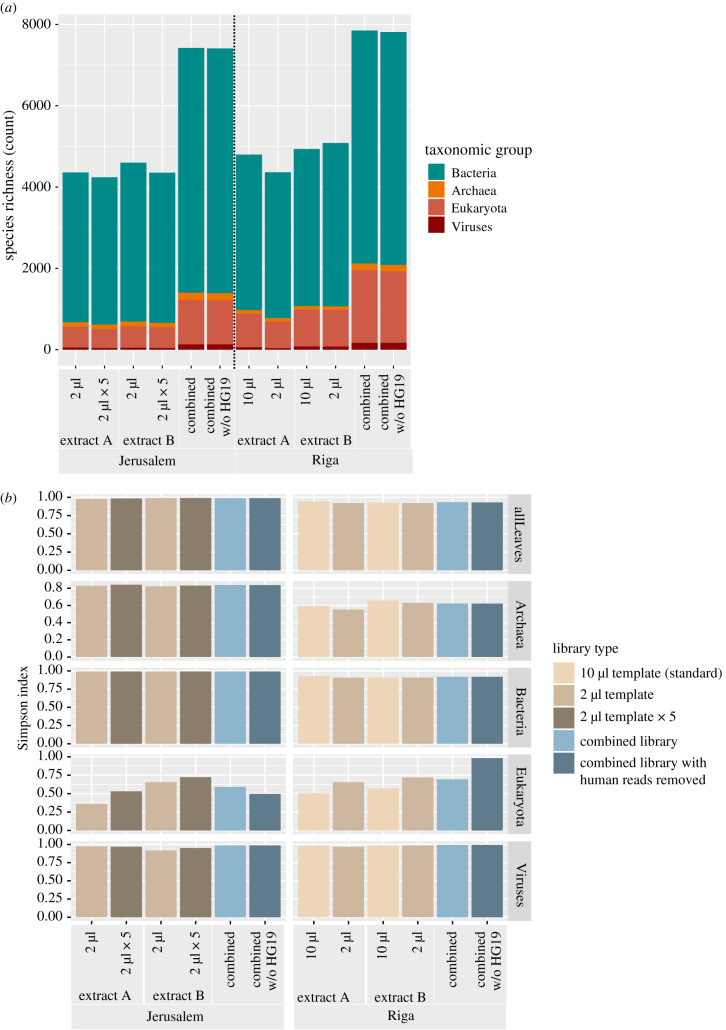
α-Diversity across all sequenced libraries. (*a*) Species richness (count of total number of species) from the metagenomic profile of each library. The bar chart is stacked by taxonomic group. Values can be found in electronic supplementary material, table S1. (*b*) Simpson's diversity indices across all libraries for each site, partitioned by taxonomic group. Simpson's index considers evenness in its formula. Indices closer to 0 have lower diversity, and indices closer to 1 have higher diversity (see formula in Methods). Values can be found in electronic supplementary material, table S2.

The extraction batch or quantity of extract used to construct each library does not appear to have played a role in the total number of species identified in the metagenome (figure 2*a*; electronic supplementary material, table S1) or the value of the Simpson's diversity index (figure 2*b*; electronic supplementary material, table S2). The Simpson's index values for species of all taxonomic groups do not differ substantially between high-template and low-template libraries, with the Riga libraries appearing slightly less diverse than those from Jerusalem. We find the computationally combined libraries, while having increased species richness, do not consistently lead to increased diversity across all taxa. As Simpson's index considers evenness, and weights common or more abundant species over rare species [35], we would likely not be missing the ‘core biome’ of the sample by taking any one of the non-combined libraries. We can infer that all species below detection in a single non-combined library from either site are represented by few reads in the dataset, and are thus difficult if not impossible to validate in downstream analyses. The possibility, therefore, remains that consideration of only single non-combined libraries could potentially prevent genetic detection of a historically or archaeologically relevant organism (e.g. a pathogen or dietary item), which might be identified via a targeted enrichment approach (e.g. [36,37]).

Despite the overall consistency of the Simpson's index values for all taxa, the diversity of different libraries is surprisingly dynamic when analyses are restricted to eukaryotic species. For Jerusalem, eukaryotic diversity is stochastic across library type. For Riga, there appears to be a pattern of increased diversity for eukaryotes in the low-template libraries compared to the standard template libraries. Furthermore, diversity in the Riga combined library increases substantially when reads mapping to the human reference genome are removed. This is likely due to an abundance of *Homo sapiens* DNA present in the Riga combined library compared to other eukaryotic organisms. Of the 76 531 reads summarized to the Eukaryota node in the Riga combined library, 43 330, or approximately 57%, were assigned to *H. sapiens.* By removing all reads that map to the HG19 human reference genome, the evenness of species representation likely increased among eukaryotes. In comparison, *H. sapiens* had reduced representation in the Jerusalem combined library, with the 8123 reads assigned to the species node making up approximately 12% of the total reads (69 110) within Eukaryota.

The most striking difference between sites in terms of α-diversity is the overall reduction in archaeal diversity in Riga compared to Jerusalem (figure 2*b*).

### (c) Estimated source contributions to bacterial and archaeal diversity

Reads summarized to the genus level were exported to a taxon table from the metagenomic profiles of the combined Jerusalem and Riga libraries with human reads removed using MEGAN6 [34] (electronic supplementary material, table S3). The taxon table was modified for compatibility with SourceTracker2 [38], a tool that produces Bayesian estimates of source contribution to a given metagenome. We modelled source contribution using datasets to represent hunter–gatherer human gut [39,40], industrialized human gut [39], sewage/wastewater [41] and soil/sediment microbiota [42–44] (electronic supplementary material, tables S4 and S5). Human reads were also removed from the source datasets.

**Figure 3.**
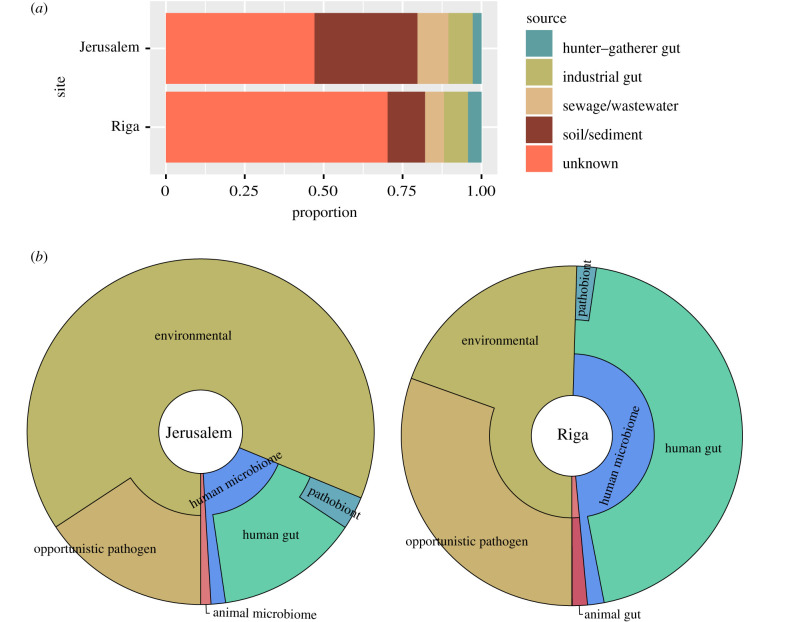
Bacterial and archaeal source analyses. (*a*) SourceTracker2 [38] was used to estimate contributions from five model sources: hunter–gatherer human gut [39,40], industrial human gut [39], sewage/wastewater [41] and soil/sediment [42–44]. Contribution was estimated using combined libraries for each site with reads mapping to the HG19 human reference genome removed. Human reads were also removed from the model source metagenomes. Estimations are based on reads summarized to the genus level within the Bacteria and Archaea nodes. (*b*) Donut plots depicting the proportion of reads summarized to bacterial and archaeal species-level nodes with at least 100 reads, grouped based on typical isolate source according to a PubMed literature survey using taxon name as the search term.

Both Jerusalem and Riga were estimated to have contributions from all sources we modelled (figure 3*a*). However, SourceTracker2 could not estimate 47% of source contribution to the Jerusalem dataset and 70% to Riga. This could be due to either a large number of taxa in Jerusalem and Riga that are shared between multiple source models or insufficiently representative source models. The gut contribution estimates are similar between Jerusalem and Riga, when the hunter–gatherer and industrialized gut categories are taken together. However, Jerusalem and Riga differ in their relative proportions of contribution by each category. This could indicate differences in the gut microbiota between the two communities or differences in the microbial dynamics of the environment surrounding the latrines. We generated a principal coordinate plot (PCoA) using the Bray–Curtis dissimilarity to further explore similarities between the latrine metagenomes and those from the model sources (see Methods; electronic supplementary material, figure S2 and table S6). The latrines occupy a liminal space between the sources, falling most closely to soil/sediment and sewage/wastewater. The overall taxonomic compositions of the samples at the phylum level show greatest similarity to soil and are largely dominated by Actinobacteria. By contrast, the gut source models are dominated by Firmicutes and the sewage/wastewater source models are dominated by Proteobacteria (electronic supplementary material, figure S3 and table S7).

In addition to using SourceTracker2 to explore the different sources of microbial diversity in our samples, we employed a manual species-sorting approach. For this analysis, we exported the MALT-generated species-level read counts from the Bacteria and Archaea nodes to taxon tables using MEGAN6 [34]. For both sites, we categorized all bacterial and archaeal species that received greater than 100 reads according to a hierarchical classification scheme reflecting their likely source environment (electronic supplementary material, tables S8 and S9). We determined the likely source environment by conducting a PubMed literature survey, using the species name as the search term. Uncultured bacteria were excluded from classification, and for recently renamed taxa, the homotypic synonyms were used as the search terms (e.g. *Mycolicibacterium smegmatis* and *Mycobacterium smegmatis*). Species were sorted into three major categories: environmental, animal-associated and human-associated. The animal-associated and human-associated categories included obligate pathogens and commensal microbiota. Species exclusively or predominantly isolated from gastrointestinal sources were specifically categorized as ‘animal gut’ or ‘human gut’ taxa (figure 3*b*). If species were considered normal constituents of human microbiota, but had the potential to cause disease, they were further categorized as pathobionts. The Riga sediments had a substantially higher representation of human gastrointestinal species than Jerusalem by this measurement.

We more closely assessed the gut microbiome signature by comparing the bacterial species present in our metagenomic analysis (i.e. species name present in taxon tables for the Jerusalem and/or Riga combined libraries) with those included in the Human Gastrointestinal Bacterial Genome and Culture Collection (HBG and HBC) [45]. Of the 153 bacterial species from the HBG list present in the Jerusalem and Riga combined libraries, 125 were present in the metagenomes from both sites (electronic supplementary material, table S10). Among the 28 species that only appeared in one of the combined libraries, none received greater than 15 assigned reads. Of the species on the HBG list, *Bifidobacterium angulatum* has the highest representation in assigned reads for both Jerusalem and Riga. *Bifidobacterium* species constituted 6 out of the top 11 most highly represented gut bacteria species in Riga. The top species in Jerusalem represented more diverse genera, including *Micrococcus*, *Anaerostipes*, *Ruminococcus* and *Escherichia*, though *Bifidobacterium* was the most abundant. The abundance of *Bifidobacterium* species in both datasets likens the community gut profiles at Jerusalem and Riga to those of modern urban populations. In multiple studies, *Bifidobacterium* has been identified as a genus enriched in industrialized populations and depleted or absent in hunter–gatherer populations [46,47] (electronic supplementary material, table S10). We also identified abundant *Treponema succinifacens* among other treponemal species (electronic supplementary material, table S6), which are typically enriched in hunter–gatherer groups and seem to have been lost in industrialized populations [39,46]. Notable absences from the latrine samples are *Alistipes putredinis* and *Eubacterium rectale*, which are among the top three most frequently identified metagenome-assembled genomes across 11 850 human gut microbiomes based on a recent study [48]. The most frequently identified species in the study, *Ruminococcus bromii*, appears to be preserved in both the Jerusalem and Riga latrines (electronic supplementary material, table S10).

### (d) Eukaryotic pathogens

We screened Jerusalem and Riga for eukaryotic pathogens through the HOPS pipeline [49], using a customized list of target taxa (electronic supplementary material, table S11). We included all taxa identified in samples from Jerusalem and Riga previously by microscopy and ELISA assay [5,6], including species within genera when identifications were made at the genus level, and close genetic relatives of the identified taxa. We also included known human-infecting parasites as listed in Ash and Orihel's *Atlas of Human Parasitology* [50]. The list was curated such that all taxon names matched those in NCBI. For each species, we included classifications on the taxonomic path up to the phylum level (e.g. for *A. lumbricoides*, we included *Ascaris*, Ascarididae, Ascaridoidea, Ascaridomorpha, Spirurina, Rhabditida, Chromadorea and Nematoda). Thus far, no *in silico* testing has been published to establish the specificity of simulated aDNA reads from eukaryotic pathogens as has been done for bacterial pathogens [49].

To have confidence in the authenticity of an aDNA alignment, one must evaluate the sequencing read's specificity to a reference sequence and the presence of damage typical of aDNA [49,51]. The former can be assessed in part through counting the number of mismatches between a sequencing read and the reference sequence to which it is aligned, called edit distance [51]. The latter can be assessed through counting specific mismatches between the read and reference sequence. Hydrolysis deaminates cytosine bases to uracil, which is read by sequencers as thymine. Cytosine-to-thymine (C > T) transitions occurring near the 5′ ends of fragments (and guanine-to-adenine (G > A) transitions near the 3′ ends) create a consistent pattern by which we may distinguish truly aDNA from modern contaminants [52–55]. HOPS provides automated assessment of DNA authenticity by calculating the distribution of edit distances of reads aligning to a given reference sequence or set of reference sequences (in the case of reads being assigned to a taxonomic node above species-level) and detecting the presence and distribution of cytosine deamination along the reads. The results of these assessments are communicated through three levels of criteria: (i) edit distance distribution of all reads, (ii) presence of C > T transitions near the 5′ ends of reads, and (iii) edit distance distribution of all reads with C > T transitions.

#### (i) Jerusalem

**Figure 4.**
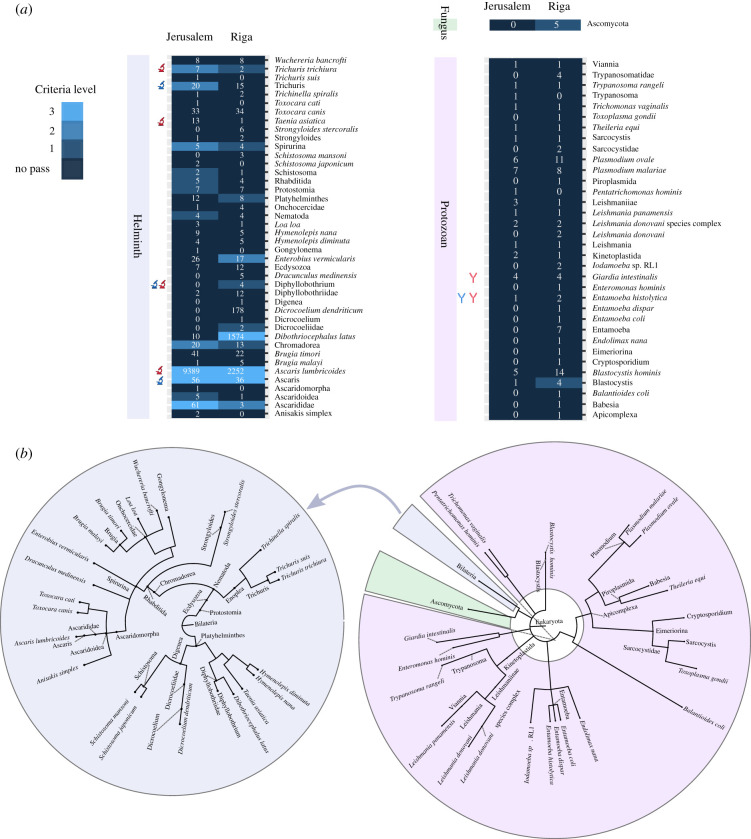
Eukaryotic parasite taxa with read alignments from latrine samples. (*a*) The taxon names to the right of the heatmap correspond to eukaryotic parasite species. All taxon nodes listed here have at least one assigned read aligning to associated sequences from the MALT results of the Jerusalem and Riga combined libraries (with reads aligning to the HG19 human reference genome removed). All taxa in this figure had assigned reads in at least one of the combined libraries, and the assigned read count (not normalized) can be found in the respective cell in the heatmap. The colour of each cell indicates the HOPS criteria passed by the alignments to that taxon. ‘No pass’—no criteria were passed, level 1—edit distance criterion was passed, level 2—damage criterion was passed, level 3—edit distance of damaged reads criterion was passed. The red and blue microscope symbols indicate the taxon was identified by microscopy in samples from Jerusalem and Riga, respectively. The red and blue antibody symbols indicate the taxon was previously identified by ELISA assay. (*b*) Radial trees representing the relationships between taxa identified in the latrine libraries, spanning multiple levels of taxonomic classification. Tabular read count results for the latrine samples can be found in electronic supplementary material, table S12, and results for negative controls can be found in electronic supplementary material, table S13.

Yeh *et al*. [5] identified the following taxa by microscopy or ELISA assay in sediments and coprolites from Jerusalem: *T. trichiura, A. lumbricoides, Taenia* sp., *Dibothriocephalus* sp., *E. histolytica* and *G. duodenalis*. Of these, we found evidence of authentic aDNA for *T. trichiura* and *A. lumbricoides.* Though *T. trichiura* passed the second HOPS threshold for authenticity, only 21 reads were assigned to the node, and nucleotide misincorporation lesions consistent with damage were present only on 2 reads. The genus node of *Trichuris* has 37 assigned reads and also passed the second level of criteria. There may be authentic *T. trichiura* DNA in the sample, and we would expect to find it given its previous microscopic identification in disaggregated sediment from Jerusalem, but at such low numbers and spread across multiple taxonomic levels, it is difficult to fully evaluate. *Ascaris lumbricoides* passed the third level of criteria according to HOPS at the species and genus levels, and several higher taxonomic levels had assigned reads which passed level two or level one criteria (e.g. Chomadorea, Nematoda; figure 4*a*; electronic supplementary material, figure S4). *Ascaris lumbricoides* accumulated sufficient assigned reads (*n* = 9389) to present a clear pattern of C > T transitions consistent with damage. Due to this convincing evidence for the presence of *A. lumbricoides*, we mapped the metagenomic data of the Jerusalem combined library directly to a single reference genome [56]. Approximately 0.26% of the Jerusalem combined library aligned to the *A. lumbricoides* sequence (table 3).

**Table 3.** Mapping statistics for *H. sapiens*, *A. lumbricoides* and *D. latus*. Mapping statistics were reported in the EAGER report table [57]. Mapping was performed in EAGER with BWA (see Methods). Mapped reads indicate the number of reads that align to the reference sequence following quality filtering and the removal of duplicate reads. Cluster factor describes the average number of times one can expect a mapped read to be duplicated, and can be used to assess redundancy in sequencing depth. Here, the presented cluster factors suggest the complexity of the libraries have not been exhausted. The mean coverage describes the mean depth of reads across the entirety of the reference sequence. Full EAGER report tables for latrine samples and negative extraction and library preparation controls can be found in electronic supplementary material, tables S14 and S15.

	sample	total sequenced reads	mapped reads	endogenous DNA (%)	cluster factor	mean coverage
*H. sapiens*	Jerusalem	16 463 260	7211	0.073	1.041	0.0002
*H. sapiens*	Riga	21 202 878	39 177	0.312	1.039	0.0009
*A. lumbricoides*	Jerusalem	16 463 260	33 863	0.264	1.068	0.0087
*A. lumbricoides*	Riga	21 202 878	1727	0.090	1.101	0.0004
*D. latus*	Riga	21 202 878	1336	0.082	1.113	0.0002

#### (ii) Riga

From the combined library for Riga, three species-level nodes passed the level two criteria: *Enterobius vermicularis*, *Dibothriocephalus latus* and *A. lumbricoides* (electronic supplementary material, figures S5 and S6). *Ascaris* and *Dibothriocephalus* sp. had been identified previously to the genus level by microscopy in the Riga sediments, and *Ascaris* presumed to be the human species of roundworm (*A. lumbricoides*) as they were found in a human latrine [6]. We proceeded to map the combined Riga libraries to single references for both *A. lumbricoides* and *D. latus*. Approximately 0.09% of reads from the combined Riga library aligned to *A. lumbricoides* and approximately 0.08% of reads aligned to *D. latus* (table 3)*.* Of the taxa identified by Yeh *et al*., there was no genetic evidence for *Oxyuris equi*; however, as only one egg was identified in the sample, and mechanical disruption of the sediment was not performed prior to DNA extraction, the absence of its detection via aDNA is not surprising. Reads aligning to *T. trichiura* passed the first threshold of HOPS authenticity criteria. Alignments to *E. vermicularis*, or human pinworm, passed the second criterion for authentication, though the species had not been identified morphologically in prior investigations. This is not unexpected as pinworm eggs are very fragile and frequently do not survive in archaeological contexts. The low number of reads, however, limits further authentication.

**Figure 5.**
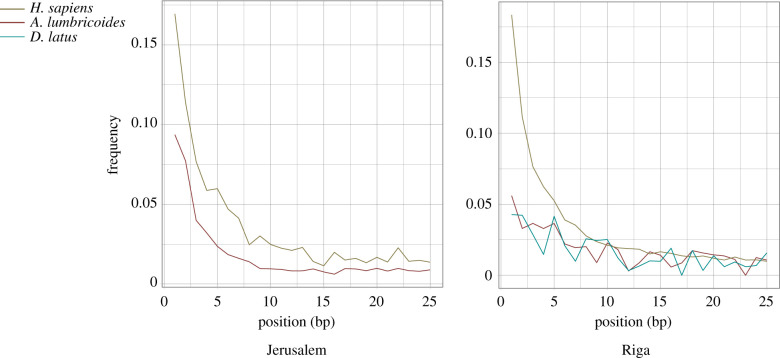
Cytosine-to-thymine mismatch plots calculated from direct alignments to *H. sapiens*, *A. lumbricoides* and *D. latus* genomes. Sequencing reads from the combined libraries were aligned using BWA (Burrows-Wheeler Aligner) and mismatch frequencies for each alignment were calculated by DamageProfiler as implemented in EAGER [57]. As the sequencing reads were extracted from latrine sediment, all alignments likely represent more than one individual of each species.

The *A. lumbricoides* and *D. latus* DNA identified in our libraries exhibited less C > T damage than the reads aligned to a human reference genome (figure 5). In the case of Jerusalem, the human DNA itself was greatly reduced compared to the *A. lumbricoides* DNA (table 3; electronic supplementary material, table S14). These phenomena could be due to protection of the DNA by the parasite eggs.

## 3. Discussion

### (a) Gut microbiome preservation and prospects for regional comparisons

The SourceTracker2 results presented here indicate (i) a large proportion of bacterial and archaeal DNA in Jerusalem and Riga does not share an affinity with any of the source models we used and (ii) Jerusalem and Riga contain approximately the same proportion of DNA from the human gut, but show slightly different proportions of the estimated human gut input between ‘hunter–gatherer’ and ‘industrialized’ microbial profiles (figure 3). Though we used model soil/sediment datasets from diverse contexts [42–44], the unknown contribution to Jerusalem and Riga could be due to our model sources not being representative of bacterial and archaeal diversity in the local soil at either archaeological site. The similarity in representation of gut bacteria between Jerusalem and Riga is intriguing. Based on our cross-referencing of species present in the libraries with the HBG list, there is no major bacterial gut species represented at one site but not the other. The SourceTracker2 analysis was performed on genus-level summarized reads, so the minor difference between estimated proportions of hunter–gatherer and industrialized gut in the Jerusalem and Riga datasets may stem from different levels of representation of certain genera. For instance, *Bifidobacterium* species are more dominant in Riga than in Jerusalem. In both sites, the presence of both abundant *Treponema* spp. and *Bifidobacterium* spp. point to these historical community gut microbiomes holding what in modern individuals would be considered a contradictory gut, being enriched in taxa that are often seen as trade-offs between more industrialized and more hunter–gatherer-based dietary habits. It is important to note, however, that these taxa represent the microbiomes of more than one individual who used the latrine.

Recent efforts to characterize the unculturable component of the human gut microbiome will likely soon lead to the expansion of databases to include a broader range of taxa [48,58]. This trend could lead to greater resolution of microbiome studies in the future as rarer and unculturable taxa are characterized, and offer the opportunity to advance our understanding of the Jerusalem and Riga data presented here. In addition, future studies of ancient latrine deposits with larger sample sizes, perhaps across geographical or cultural gradients, could contribute to our understanding of gut microbiome variation in the past.

In terms of the overall bacterial and archaeal content of the latrine samples in comparison with the model sources used for the SourceTracker2 analysis, we found the Jerusalem and Riga combined libraries clustered closely with each other in a principal coordinate analysis, and of the model sources they clustered most closely with the soil/sediment dataset (electronic supplementary material, figure S2). Curiously, we also observed that the global sewage/wastewater dataset aligns closely with industrial and hunter–gatherer gut microbiome along PC1, but forms its own cluster in multiple dimensions (electronic supplementary material, figure S2a,b). Importantly, the sewage/wastewater and latrine datasets considered here do not appear to be mere aggregates of gut microbiota, but rather have formed based on the contribution of microbial contents specific to their depositional contexts. We furthermore note that the depositional context in question for the latrine samples is one transformed by hundreds of years of burial.

### (b) Eukaryotic parasites: the promise and limitations of ancient metagenomics

Of the eukaryotic parasites previously morphologically identified in sediment and coprolites from Jerusalem and Riga, confident genetic identifications of *A. lumbricoides* in Jerusalem and *A. lumbricoides* and *D. latus* in Riga were possible. A small quantity of reads from each library was assigned to the other previously identified eukaryotic parasites (or closely related taxa) with the exception of *Oxyuris equi*, though these were not sufficient to allow authentication (figure 5)*.* The absence of *O. equi* from the metagenomic profiles generated here is unsurprising, given that only one egg was identified in the latrine sediment [6]. *Enterobius vermicularis* received assigned reads from Jerusalem and Riga, and the 17 reads from Riga passed the second threshold of authentication criteria determined in HOPS, meaning the assigned reads sufficiently fit the preferred edit distance distribution from the reference sequence, and some reads contained C > T damage expected in authentic aDNA [49]. This is the only organism that was not previously identified for which assigned reads passed the second criteria. Its presence would be expected in metagenomic datasets including human faecal material as *E. vermicularis* and other species with *Enterobius* maintain reservoirs in humans and non-human primates [21,24]. Previous research has found aDNA signatures of this fragile parasite egg in archaeological sediment despite lack of preservation of intact eggs being visible on microscopy [17].

The differences in identified species between the former palaeoparasitology analyses and this genetic analysis are likely due to the following reasons: (i) samples and sampling methods; (ii) the metagenomic approach; and (iii) differences in the availability of reference sequences between different parasite taxa. For the Jerusalem latrine, 0.2 g of loose sediment and 12 semi-mineralized coprolites were analysed using microscopy, and 1 g for ELISA [5]; for the Riga latrine, 1 g of loose sediment was analysed using microscopy and ELISA [6]. In the analysis presented here, 100 mg of loose sediment was used in total for each site to build all metagenomic representations, and the Jerusalem coprolites were not studied. The extraction protocol used in this study was designed to particularly preserve short (presumably ancient) fragments of DNA from small samples of less than 100 mg of material. The quantity of material analysed differs greatly between the genetic, microscopy and ELISA methods employed and referenced here, and the reduced amounts used for DNA evaluation—up to 10-fold less than what has been used for other analytical techniques—could account for some of the differences we see in the species identifications between the methods. We did not implement mechanical disruption prior to extraction, which may have limited our ability to extract DNA from helminth eggs, though the strong signal from *A. lumbricoides* suggests that this was not prohibitive. Prior genetic investigations into ancient eukaryotic parasites (largely dominated by *A. lumbricoides*) have been targeted either through the direct extraction and sequencing of identified and filtered parasite eggs or through amplicon sequencing of specific genes belonging to a single species or small selection of taxa [10,13–15,17–19,59]. Here, we extracted aliquots of the bulk sample directly, foregoing filtration of the sediments for eggs to both avoid potential contamination during egg filtration and to gain a faithful metagenomic approximation of the biological community in the latrine. These measures support the integrity of our data in exchange for high read abundances from all parasites present in the latrine. Furthermore, we did not limit this analysis by selective amplification. An additional reason for why we see weaker genetic representation from some of the taxa identified in the prior studies for both sites could result from unbalanced representations of many eukaryotic parasite taxa in the NCBI nucleotide database. It is plausible that DNA fragments of eukaryotic parasites in the metagenomic libraries used here were not identifiable using current databases, because they belong to portions of the genome that have not yet been assembled.

Given the extremely low coverage for even the most strongly represented eukaryotic pathogens in this dataset, what is the benefit of a metagenomics approach to palaeoparasitology? Morphology is undoubtedly the most reliable means of establishing the presence of helminths in a population through analysis of palaeofaeces or latrine sediments, because there is little room for ambiguity regarding identifications of species whose morphology is well established. However, fine-grain taxonomic identification is not possible for some groups where different species of the same genus produce eggs of identical morphology (e.g. *Taenia* spp. and *Entamoeba* spp.) and taphonomic processes may prevent identification of protozoa and fragile helminth eggs such as hookworm and pinworm [10,25]. Genetic data may offer such fine-grain taxonomic identification for helminths and protozoa. In this case, a metagenomic sequencing coupled with a lowest common ancestor taxonomic binning approach [19], as opposed to a marker gene or amplicon approach [60], may offer greater possibility of species identifications from degraded, fragmentary DNA. Following relatively unbiased identification of an organism, coverage may be increased through either deeper sequencing or genomic enrichment. The resulting data could then be thoroughly authenticated and used to elucidate the evolutionary history of a given parasite. However, the full promise of palaeoparasitology by metagenomics to answer high-resolution questions of parasite phylogeny and evolution cannot be fulfilled until more complete and reliable modern reference databases are available. For instance, differentiating between *D. latus* and *Dibothriocephalus dendriticum,* two closely related but distinct species of fish tapeworm, with low coverage metagenomic data would likely bias for *D. latus* due to the increased number of full genome entries available. Groups such as the International Helminth Genomes Consortium are working to fill existing database gaps [56]. Such efforts are instrumental for realizing the full benefit of the consilient approaches to ancient parasitology explored here.

### (c) Template volume did not impact metagenomic α-diversity

Despite the inhibition phenomenon we detected through our quantitation of starting template number in the pre-indexed libraries (table 1), we did not observe an impact on the α-diversity measurements of the resulting metagenomes. We can, therefore, infer that the 10 µl library preparation for the Riga samples would not result in a meaningful decrease in diversity (as calculated with the Simpson's index) for all taxa together—Bacteria, Archaea or Viruses. However, for the Riga site, we do see an increase in Eukaryotic diversity for the 2 µl libraries over the 10 µl libraries. The mechanism behind this signature is unclear. In our example, no discernable pattern emerged regarding the relationship between template volume and α-diversity for the 2 µl and 2 µl × 5 libraries. It is unclear whether this lack of relationship stems from inhibition or sufficiency of the low-template volume to produce a representative metagenome for the sample; α-diversity measurements could differ when similar approaches to library construction are followed for highly complex, or more molecularly diverse, extracts and further exploration of the phenomenon of inhibition in other contexts is warranted. However, the relatively low cluster factors (less than 2) we report in our mapping statistics for *A. lumbricoides* and *D. latus*, which provide an estimation of how many times individual reads are seen in a given dataset based on the duplication rate of mapped reads, indicate the combined sequencing depth for each site has not exhausted the complexity of the libraries (table 3). A cluster factor of 2 or greater would indicate that every molecule has been sequenced at least twice on average, hence sequencing to this depth would be advantageous for evaluating total metagenomic diversity. Regardless, the diversity in terms of species richness for the libraries considered here appears not to be impacted by reductions in sequencing depth.

## 4. Conclusion

This study demonstrates the preservation of DNA representative of human intestinal contents in ancient latrines from the Medieval Period. Impressively, DNA preservation extends beyond molecules protected within resilient physical structures such as parasite eggs: signals of gut flora are detectable, though microbial content is heavily influenced by depositional context. Therefore, the archaeological latrine samples considered here do not represent direct snapshots of the microbial community in ancient waste management systems, but rather depict microbial profiles that have been altered by many years of exposure to environmental sources. While in use, a properly functioning latrine permits decomposition of most organic components, which will necessarily disrupt structure of the host microbial community through degradation of some bacterial species and proliferation of others. Observations from coprolites that formed via rapid desiccation are presumed to differ, where blooms of environmental bacteria and disruption of community structure via overgrowth are less likely. Regardless, DNA preservation in latrines does occur, as demonstrated by our identification of well-known gut microbiota and several parasite species. This work highlights the value of ancient latrines as sources of biomolecular information, and the benefits that come from the marriage of complementary analytical approaches to develop an understanding of past human health.

## 5. Material and methods

### (a) Sampling and DNA extraction

Samples of latrine sediment were taken at the time of excavation by archaeologists at the sites (Jerusalem and Riga). Each sample was taken using a clean spoon and placed immediately into a small plastic bag that was sealed, without touching human skin. This bag was then triple-bagged to prevent any contamination from outside, or leakage of samples from inside. The samples were then sent to the Ancient Parasites Laboratory at Cambridge for analysis. Subsamples were removed while wearing laboratory gloves in a Class 2 Microbiological Safety Cabinet, prior to preparation for microscopy (0.2 g samples) and ELISA (1 g samples) [5,6]. The rest of the sample remained protected from the environment, triple-bagged. After completion of the parasite analysis, 3 g subsamples from the original bags were removed in the microbiological safety cabinet while wearing laboratory gloves, placed in fresh bags, sealed and triple-bagged. These 3 g samples were then sent to the Max Planck Institute for Science of Human History (MPI-SHH) in Jena, Germany. While it would have been helpful if samples of sediment were available from outside the latrine dating from the same time period that the latrine was in use, in order to determine the normal soil microbiome, such samples were not taken at the time of excavation.

Further sample preparation, DNA extraction, Illumina library preparation and sequencing were conducted at the dedicated aDNA clean room (pre-PCR) and post-PCR facilities of the MPI-SHH. Two spatially separate portions of approximately 50–80 mg of sediment were taken from each sample, and a total of four extractions were performed. Extractions were performed using a protocol optimized for use with archaeological bone [61], and later adapted for sediment [43]. Each subsample was immersed in 1 ml of 0.5 M EDTA and rotated overnight at room temperature. After decalcification, the sample was centrifuged and the supernatant was purified. Each sample was added to 10 ml of 5 M guanidine-hydrochloride binding buffer and 400 µl sodium acetate, mixed by inversion and transferred to a High Pure Extender column from the High Pure Viral Nucleic Acid Large Volume kit (Roche). After centrifugation of the binding buffer solution, the column was transferred to a 1.5 ml collection tube and given a dry spin to ensure the silica membrane was free of any excess buffer. Two washing steps were performed with the kit wash buffer. Elution was performed using 100 µl of a 10 mM tris–hydrochloride, 1 mM EDTA (pH 8.0) and 0.05% Tween-20 buffer (TET) for all extracts. The eluate from the Jerusalem extracts had a dark-brown tinge, and that of the Riga extracts had a very slight pale-yellow tinge, indicating impurities in the extract. Two negative controls were included in the extraction batch to detect reagent- and process-derived contamination.

### (b) Library preparation

#### (i) Initial screening and inhibition test

Construction of double-stranded Illumina libraries was performed according to a modified established protocol [62]. DNA overhangs were removed and filled-in with a 50 µl reaction including 5 µl of NEB Buffer 2 (New England Biolabs), 2 µl dNTP mix (2.5 mM), 4 µl BSA (10 mg ml^−1^), 5 µl ATP (10 mM), 2 µl T4 polynucleotide kinase and 0.4 µl T4 polymerase. Four libraries (Jerusalem A1, Jerusalem B1, Riga A1 and Riga B1) were constructed using 10 µl of DNA extract and 21.6 µl H_2_O. Two additional libraries (Jerusalem A2 and Jerusalem B2) were constructed using 2 µl of DNA extract and 29.6 µl H_2_O in response to the colouring of the Jerusalem extracts. We wished to test if chemicals remaining in the Jerusalem extracts would inhibit downstream PCR steps by lowering the template volume and comparing copy number calculations after quantitating the non-indexed libraries with a real-time qPCR assay (Lightcycler 480 Roche; see below). Following the incubation of the initial overhang repair reactions, they were purified over MinElute columns (Qiagen) and eluted in 18 µl TET. Universal Illumina adapters were ligated to the repaired fragments with a reaction of 20 µl Quick Ligase Buffer, 1 µl Illumina adapter mix (0.25 µM) and 1 µl of Quick Ligase. The reaction was then purified as described above and eluted in 20 µl of TET. Adapter fill-in was performed in 40 µl reactions, including 4 µl Thermopol buffer, 2 µl dNTP mix (2.5 mM) and 2 µl Bst polymerase. Following incubation at 37°C for 20 min, the enzyme was heat-deactivated with an additional 20 min incubation at 80°C. Two negative controls were included in the workflow to track contamination specifically in the library preparation reagents and process. The resulting pre-indexed libraries were quantitated with qPCR, at which point inhibition in the Jerusalem libraries was evaluated (see Results). The Jerusalem libraries built with 2 µl of template (Jerusalem A2 and B2) were carried forward for further processing.

Later, five pre-indexed libraries were constructed from each extract for Jerusalem using 2 µl of template each (Jerusalem A3.1, A3.2, A3.3, A3.4, A3.5, B3.1, B3.2, B3.3, B3.4 and B3.5) and one pre-indexed library was constructed from each extract for Riga using 2 µl of template to test whether we had an inhibition effect in the qPCR results for Riga as well.

#### (ii) Indexing, amplification and sequencing

All pre-indexed libraries that were carried forward for sequencing were double indexed with unique pairs of indices in 100 µl reactions containing fewer than 1.5 × 10^10^ DNA fragments. All libraries referred to as Jerusalem A3 were given the same indices. All libraries referred to as Jerusalem B3 were given the same indices. Each reaction included 10 µl PfuTurbo buffer, 1 µl PfuTurbo Cx Hotstart DNA polymerase (Agilent), 1 µl dNTP mix (25 mM), 1.5 µl BSA (10 mg ml^−1^) and 2 µl of each indexing primer (10 µM). The master mix was prepared in a pre-PCR clean room and transported to a separate laboratory for amplification. All reactions belonging to the same library were purified over MinElute columns. The indexed Jerusalem A3 sublibraries and Jerusalem B3 sublibraries were combined during purification. Indexed libraries were eluted in 50 µl of TET. The indexing efficiency was assessed using a qPCR assay targeting the IS5 and IS6 sequences in the indexing primers. Approximately one-third of each indexed library was amplified with Herculase II Fusion DNA Polymerase (Agilent). The products were MinElute purified and quantified using an Agilent Tape Station D1000 Screen Tape kit. Jerusalem A2, Jerusalem B2, Riga A1, and Riga A2 were sequenced on an Illumina NextSeq 500 using a paired-end, 75-cycle high-output kit to depths of 5–6 million paired reads each. Jerusalem A3, Jerusalem B3, Riga A2 and Riga B2 were sequenced on an Illumina NextSeq 500 using a single-end, 75-cycle high-output kit to depths of 5–6 million reads each. Negative controls were sequenced separately to avoid index cross-talk to a depth of approximately 1 million reads each.

### (c) Computational methods

Find a flow-chart illustrating the main computational analysis pipelines used in this study in electronic supplementary material, figure S1.

#### (i) Pre-processing and mapping to HG19

We pre-processed and mapped de-multiplexed reads for each library using the EAGER pipeline [57]. Preliminary adapter-removal and read filtering was performed using AdapterRemoval, enforcing a minimum read length of 30 and a minimum base quality of 20. Paired reads for Jerusalem A2, Jerusalem B2, Riga A1 and Riga B1 were also merged. All reads were mapped to the HG19 human reference genome using BWA [63] (as implemented in EAGER) with a quality filter of 30, difference parameter (−*n*) of 0.01 and deactivated seed length filter (−*l*). We performed this mapping as a standard exploratory analysis of human DNA preservation. In addition, we concatenated the fastq files from libraries belonging to the same site and mapped these combined files to the HG19 reference genome using BWA outside of the EAGER pipeline in order to generate a fastq file with all human reads extracted. We did this to test the impact of removing human reads on the metagenomic profile of the latrines, as we expect some genetic sequences from human-associated parasites to be contaminated with human DNA. For this analysis, we applied −*n* 0.1 and −*l* 32.

#### (ii) Metagenomic profiling and pathogen detection

We executed a broad screening of the total genetic content of the shotgun-sequenced libraries using the HOPS pipeline [33] which joins the taxonomic binning tool MALT [32] with MaltExtract and a post-processing script which produces a heatmap of putatively authentic taxon identifications based on edit distance from a reference sequence and the presence of damaged reads. As input, we used fastq files from the pre-processing step above, including the original fastq files representing individual libraries, the combined fastq files and the combined fastq files with human-mapped reads extracted. For MALT (v. 040), we used the NCBI full nucleotide database (‘nt’, October 2017) with a 90% identity threshold. To analyse the overall bacterial and archaeal content of the latrine samples in comparison to metagenomic datasets from known sources (electronic supplementary material, table S5), we generated a series of read-depth normalized taxon tables with MEGAN6 [34] from the raw MALT results. The model sources represented hunter–gatherer human gut [39,40], industrialized human gut [39], sewage/wastewater [41] and sediment [42–44]. To explore the relative proportions of different phyla, we generated a taxon table of reads summarized to phylum nodes, normalized across a subset of source models and the latrine samples (electronic supplementary material, figure S3 and table S7). We further explored the relationship between the latrine metagenomes and those from known sources through a PCoA generated using a Bray–Curtis taxonomic distance matrix based on species-level assigned reads (electronic supplementary material, figure S2 and table S6). The dissimilarity matrix was calculated using the *vegan* package [64] and plotted using the *ggplot2* package [65] in R. For the SourceTracker2 analysis (figure 2), we extracted a taxon table of reads summarized to the genus level among Bacteria and Archaea (electronic supplementary material, table S3) and reformatted it to be compatible with the tool. To accompany this, we conducted a non-inferential approach in which we generated donut plots to explore the total metagenomic profile for the combined libraries (excluding human reads) for Jerusalem and Riga using Krona in terms of the quantity of reads assigned to individual species which were classified according to likely source (electronic supplementary material, tables S8 and S9). The nested classification scheme divided the species belonging to the domains Bacteria and Archaea, then into the categories of ‘environmental’, ‘human microbiome’ and ‘animal microbiome’. The environmental taxa were then sorted according to whether or not they were known opportunistic pathogens. The ‘human’ and ‘animal’ microbiome taxa were classified into whether they were specifically associated with the gastrointestinal system and whether they were known to occasionally cause disease states as pathobionts. Classifications were made through evaluation of PubMed literature available for each species present in the two latrine metagenomes, using the species' Latin binomial as the search parameter. Uncultured and unnamed bacteria were excluded from this analysis due to a lack of published metadata.

To identify and authenticate particular pathogenic and parasitic taxa, we curated a taxonomy list designed to target (i) bacterial and viral pathogens of broad interest, (ii) human-associated eukaryotic parasites [50] and (iii) parasites previously identified by microscopy in the Jerusalem and Riga latrine deposits [5,6]. The taxon names for the eukaryotic pathogens were checked against the NCBI database to ensure nomenclature matched between the taxon list and the database (electronic supplementary material, table S11). Taxonomic levels up to phylum were included in the list for Eukaryotic pathogens (e.g. for the target species *A. lumbricoides*, we included *Ascaris*, Ascarididae, Ascaridoidea, Ascaridomorpha, Spirurina, Rhabditida, Chromadorea, Nematoda, Ecdysozoa and Protostomia).

#### (iii) Community diversity

We calculated species richness and Simpson's index of diversity for each library with species-level taxon tables extracted using MEGAN6 [34]. α-Diversity calculations were performed with the *vegan* package in R [64]. The following taxon tables were extracted: all terminal leaves (in all domains of life) at species level, leaves below the Bacteria and Archaea nodes at species level, leaves below the Eukaryota node at species level, leaves below the Viruses node at species level, all leaves at genus level, leaves below the cellular organisms node at genus level, leaves below the Bacteria and Archaea nodes at genus level, leaves below the Eukaryota node at genus level and leaves below the Viruses node at genus level. Species richness was calculated using the ‘specnumber’ function in the *vegan* R package [64] (electronic supplementary material, table S1). Here, we express Simpson's index of diversity according to the formula used by the *vegan* package in terms of *D*, where *p* is the proportion of abundances between species *i* and the total abundance in the community. In this case, total reads aligning to either a species- or genus-level node are the abundances

$$D=\;\sum p_{i}^{2}.$$

The resulting value *D* is then subtracted from 1 to yield diversity values between 0 and 1, with values closer to 0 indicating less diversity and values closer to 1 indicating more diversity. Simpson's diversity results were reached by using the ‘diversity’ function with the ‘simpson’ choice as implemented in the *vegan* R package [64] (electronic supplementary material, table S2).

#### (iv) Mapping to *Ascaris lumbricoides* and *Dibothriocephalus latus*

Following HOPS analysis, we mapped the combined libraries for Jerusalem and Riga to genomic assemblies for *A. lumbricoides* and *D. latus* with BWA [63] as implemented in the EAGER pipeline (v. 1.92) [57]. As the reference for *A. lumbricoides*, we used the genome available from WormBase as of 13 February 2019 [56,66]. As the reference for *D. latus*, we used the full genomic assembly available under accession number PRJEB1206 [67]. For both organisms, we used the following mapping parameters: −*l* 16, −*n* 0.01 and −*q* 37. Duplicates were removed with MarkDuplicates, and damage profiling was performed with mapDamage [54] (electronic supplementary material, tables S14 and S15).

## Supplementary Material

Supplementary figures

## Supplementary Material

Supplementary tables

## Data accessibility

The shotgun sequencing data generated as part of this study can be accessed on the NCBI BioProject database under accession number PRJNA640385. Accession numbers for the model source datasets can be found in electronic supplementary material, table S5.

## References

[1] Fugassa MH, Beltrame MO, Sardella NH, Civalero MT, Aschero C 2010 Paleoparasitological results from coprolites dated at the Pleistocene–Holocene transition as source of paleoecological evidence in Patagonia. J. Archaeol. Sci. 37, 880–884. (10.1016/j.jas.2009.11.018)

[2] Han E-T, Guk S-M, Kim J-L, Jeong H-J, Kim S-N, Chai J-Y 2003 Detection of parasite eggs from archaeological excavations in the Republic of Korea. Mem. Inst. Oswaldo Cruz 98, 123–126. (10.1590/S0074-02762003000900018) 12687771

[3] Mitchell PD, Anastasiou E, Syon D 2011 Human intestinal parasites in crusader Acre: evidence for migration with disease in the medieval period. Int. J. Paleopathol. 1, 132–137. (10.1016/j.ijpp.2011.10.005) 29539328

[4] Reinhard KJ, Hevly RH, Anderson GA 1987 Helminth remains from prehistoric Indian coprolites on the Colorado Plateau. J. Parasitol. 73, 630 (10.2307/3282147) 3298603

[5] Yeh H-Y, Prag K, Clamer C, Humbert J-B, Mitchell PD 2015 Human intestinal parasites from a Mamluk Period cesspool in the Christian quarter of Jerusalem: potential indicators of long distance travel in the 15th century AD. Int. J. Paleopathol. 9, 69–75. (10.1016/j.ijpp.2015.02.003) 29539442

[6] Yeh H-Y, Pluskowski A, Kalējs U, Mitchell PD 2014 Intestinal parasites in a mid-14th century latrine from Riga, Latvia: fish tapeworm and the consumption of uncooked fish in the medieval eastern Baltic region. J. Archaeol. Sci. 49, 83–89. (10.1016/j.jas.2014.05.001)

[7] Gonçalves MLC, Araújo A, Duarte R, da Silva JP, Reinhard K, Bouchet F, Ferreira LF 2002 Detection of *Giardia duodenalis* antigen in coprolites using a commercially available enzyme-linked immunosorbent assay. Trans. R. Soc. Trop. Med. Hyg. 96, 640–643. (10.1016/S0035-9203(02)90337-8) 12625140

[8] Le Bailly M, Gonçalves ML, Harter-Lailheugue S, Prodéo F, Araujo A, Bouchet F 2008 New finding of *Giardia intestinalis* (Eukaryote, Metamonad) in Old World archaeological site using immunofluorescence and enzyme-linked immunosorbent assays. Mem. Inst. Oswaldo Cruz 103, 298–300. (10.1590/S0074-02762008005000018) 18545853

[9] Mitchell PD, Stern E, Tepper Y 2008 Dysentery in the crusader kingdom of Jerusalem: an ELISA analysis of two medieval latrines in the city of Acre (Israel). J. Archaeol. Sci. 35, 1849–1853. (10.1016/j.jas.2007.11.017)

[10] Côté NML et al. 2016 A new high-throughput approach to genotype ancient human gastrointestinal parasites. PLoS ONE 11, e0146230 (10.1371/journal.pone.0146230) 26752051 PMC4709038

[11] Cleeland LM, Reichard MV, Tito RY, Reinhard KJ, Lewis CM Jr 2013 Clarifying prehistoric parasitism from a complementary morphological and molecular approach. J. Archaeol. Sci. 40, 3060–3066. (10.1016/j.jas.2013.03.010) 23645967 PMC3640563

[12] Flammer PG et al. 2018 Molecular archaeoparasitology identifies cultural changes in the Medieval Hanseatic trading centre of Lübeck. Proc. R. Soc. B 285, 20180991 (10.1098/rspb.2018.0991) PMC6191690 30282648

[13] Guhl F, Jaramillo C, Vallejo GA, Yockteng R, Cardenas-Arroyo F, Fornaciari G, Arriaza B, Aufderheide AC 1999 Isolation of *Trypanosoma cruzi* DNA in 4,000-year-old mummified human tissue from northern Chile. Am. J. Phys. Anthropol. 108, 401–407. (10.1002/(SICI)1096-8644(199904)108:4<401::AID-AJPA2>3.0.CO;2-P) 10229385

[14] Iñiguez AM, Reinhard K, Carvalho Gonçalves ML, Ferreira LF, Araújo A, Paulo Vicente AC 2006 SL1 RNA gene recovery from *Enterobius vermicularis* ancient DNA in pre-Columbian human coprolites. Int. J. Parasitol. 36, 1419–1425. (10.1016/j.ijpara.2006.07.005) 16950265

[15] Iñiguez AM, Reinhard KJ, Araújo A, Ferreira LF, Vicente ACP 2003 *Enterobius vermicularis*: ancient DNA from north and south American human coprolites. Mem. Inst. Oswaldo Cruz 98, 67–69. (10.1590/S0074-02762003000900013) 12687766

[16] Loreille O, Roumat E, Verneau O, Bouchet F, Hänni C 2001 *Ancient* DNA from *Ascaris*: extraction amplification and sequences from eggs collected in coprolites. Int. J. Parasitol. 31, 1101–1106. (10.1016/S0020-7519(01)00214-4) 11429174

[17] Maicher C, Hoffmann A, Côté NM, Palomo Pérez A, Saña Segui M, Le Bailly M. 2017 Paleoparasitological investigations on the Neolithic lakeside settlement of La Draga (Lake Banyoles, Spain). The Holocene 27, 0959683617702236. (10.1177/0959683617702236)

[18] Oh CS, Seo M, Lim NJ, Lee SJ, Lee E-J, Lee SD, Shin DH 2010 Paleoparasitological report on *Ascaris* aDNA from an ancient East Asian sample. Mem. Inst. Oswaldo Cruz 105, 225–228. (10.1590/S0074-02762010000200020) 20428686

[19] Søe MJ et al. 2018 Ancient DNA from latrines in Northern Europe and the Middle East (500 BC–1700 AD) reveals past parasites and diet. PLoS ONE 13, e0195481 (10.1371/journal.pone.0195481) 29694397 PMC5918799

[20] Wiscovitch-Russo R, Rivera-Perez J, Narganes-Storde YM, García-Roldán E, Bunkley-Williams L, Cano R, Toranzos GA 2020 Pre-Columbian zoonotic enteric parasites: an insight into Puerto Rican indigenous culture diets and life styles. PLoS ONE 15, e0227810 (10.1371/journal.pone.0227810) 31999735 PMC6992007

[21] Araújo A, Reinhard K, Ferreira LF, Pucu E, Chieffi PP 2013 Paleoparasitology: the origin of human parasites. Arq. Neuro-Psiquiatr. 71, 722–726. (10.1590/0004-282X20130159) 24141513

[22] Araújo A, Reinhard KJ, Ferreira LF, Gardner SL 2008 Parasites as probes for prehistoric human migrations? Trends Parasitol. 24, 112–115. (10.1016/j.pt.2007.11.007) 18262843

[23] Araújo A, Ferreira LF 2000 Paleoparasitology and the antiquity of human host-parasite relationships. Mem. Inst. Oswaldo Cruz 95, 89–93. (10.1590/S0074-02762000000700016) 11142733

[24] Mitchell PD 2013 The origins of human parasites: exploring the evidence for endoparasitism throughout human evolution. Int. J. Paleopathol. 3, 191–198. (10.1016/j.ijpp.2013.08.003) 29539455

[25] Mitchell PD 2015 Human parasites in medieval Europe: lifestyle, sanitation, and medical treatment. Adv. Parasitol. 90, 389–420. (10.1016/bs.apar.2015.05.001) 26597073

[26] Perry GH 2014 Parasites and human evolution. Evol. Anthropol. Issues News Rev. 23, 218–228. (10.1002/evan.21427) 25627083

[27] Cano RJ, Rivera-Perez J, Toranzos GA, Santiago-Rodriguez TM, Narganes-Storde YM, Chanlatte-Baik L, García-Roldán E, Bunkley-Williams L, Massey SE 2014 Paleomicrobiology: revealing fecal microbiomes of ancient indigenous cultures. PLoS ONE 9, e106833 (10.1371/journal.pone.0106833) 25207979 PMC4160228

[28] Santiago-Rodriguez TM, Fornaciari G, Luciani S, Dowd SE, Toranzos GA, Marota I, Cano RJ 2015 Gut microbiome of an 11th century A.D. pre-Columbian Andean mummy. PLoS ONE 10, e0138135 (10.1371/journal.pone.0138135) 26422376 PMC4589460

[29] Tito RY et al. 2008 Phylotyping and functional analysis of two ancient human microbiomes. PLoS ONE 3, e3703 (10.1371/journal.pone.0003703) 19002248 PMC2577302

[30] Tito RY et al. 2012 Insights from characterizing extinct human gut microbiomes. PLoS ONE 7, e51146 (10.1371/journal.pone.0051146) 23251439 PMC3521025

[31] King CE, Debruyne R, Kuch M, Schwarz C, Poinar HN 2009 A quantitative approach to detect and overcome PCR inhibition in ancient DNA extracts. BioTechniques 47, 941–949. (10.2144/000113244) 20041847

[32] Vågene ÅJ et al. 2018 *Salmonella enterica* genomes from victims of a major sixteenth-century epidemic in Mexico. Nat. Ecol. Evol. 2, 520–528. (10.1038/s41559-017-0446-6) 29335577

[33] Hübler R, Key FM, Warinner C, Bos KI, Krause J, Herbig A 2019 HOPS: automated detection and authentication of pathogen DNA in archaeological remains. Genome Biol. 20, 280 (10.1186/s13059-019-1903-0) 31842945 PMC6913047

[34] Huson DH, Beier S, Flade I, Górska A, El-Hadidi M, Mitra S, Ruscheweyh H-J, Tappu R 2016 MEGAN community edition—interactive exploration and analysis of large-scale microbiome sequencing data. PLoS Comput. Biol. 12, e1004957 (10.1371/journal.pcbi.1004957) 27327495 PMC4915700

[35] Xia Y, Sun J, Chen D-G 2018 Statistical analysis of microbiome data with r. New York, NY: Springer.

[36] Schuenemann VJ et al. 2011 Targeted enrichment of ancient pathogens yielding the pPCP1 plasmid of *Yersinia pestis* from victims of the Black Death. Proc. Natl Acad. Sci. USA 108, E746–E752. (10.1073/pnas.1105107108) 21876176 PMC3179067

[37] Bos KI et al. 2011 A draft genome of *Yersinia pestis* from victims of the Black Death. Nature 478, 506–510. (10.1038/nature10549) 21993626 PMC3690193

[38] Knights D, Kuczynski J, Charlson ES, Zaneveld J, Mozer MC, Collman RG, Bushman FD, Knight R, Kelley ST 2011 Bayesian community-wide culture-independent microbial source tracking. Nat. Methods 8, 761–763. (10.1038/nmeth.1650) 21765408 PMC3791591

[39] Obregon-Tito AJ et al. 2015 Subsistence strategies in traditional societies distinguish gut microbiomes. Nat. Commun. 6, 6505 (10.1038/ncomms7505) 25807110 PMC4386023

[40] Rampelli S et al. 2015 Metagenome sequencing of the Hadza hunter-gatherer gut microbiota. Curr. Biol. 25, 1682–1693. (10.1016/j.cub.2015.04.055) 25981789

[41] Hendriksen RS et al. 2019 Global monitoring of antimicrobial resistance based on metagenomics analyses of urban sewage. Nat. Commun. 10, 1–12. (10.1038/s41467-019-08853-3) 30850636 PMC6408512

[42] Orellana LH, Chee-Sanford JC, Sanford RA, Löffler FE, Konstantinidis KT 2017 Year-round shotgun metagenomes reveal stable microbial communities in agricultural soils and novel ammonia oxidizers responding to fertilization. Appl. Environ. Microbiol. 84, e01646-17 (10.1128/AEM.01646-17) PMC5752871 29101194

[43] Slon V et al 2017 Neandertal and Denisovan DNA from Pleistocene sediments. Science 356, 605–608. (10.1126/science.aam9695) 28450384

[44] Tripathi BM, Moroenyane I, Sherman C, Lee YK, Adams JM, Steinberger Y 2017 Trends in taxonomic and functional composition of soil microbiome along a precipitation gradient in Israel. Microb. Ecol. 74, 168–176. (10.1007/s00248-017-0931-0) 28074247

[45] Forster SC et al. 2019 A human gut bacterial genome and culture collection for improved metagenomic analyses. Nat. Biotechnol. 37, 186 (10.1038/s41587-018-0009-7) 30718869 PMC6785715

[46] Angelakis E et al. 2019 *Treponema* species enrich the gut microbiota of traditional rural populations but are absent from urban individuals. New Microb. New Infect. 27, 14–21. (10.1016/j.nmni.2018.10.009) PMC6276622 30555706

[47] Schnorr SL et al. 2014 Gut microbiome of the Hadza hunter-gatherers. Nat. Commun. 5, ncomms4654 (10.1038/ncomms4654) PMC3996546 24736369

[48] Almeida A, Mitchell AL, Boland M, Forster SC, Gloor GB, Tarkowska A, Lawley TD, Finn RD 2019 A new genomic blueprint of the human gut microbiota. Nature 568, 499 (10.1038/s41586-019-0965-1) 30745586 PMC6784870

[49] Huebler R, Key FM, Warinner C, Bos KI, Krause J, Herbig A 2019 HOPS: automated detection and authentication of pathogen DNA in archaeological remains. bioRxiv 534198. (10.1101/534198). PMC6913047 31842945

[50] Ash LR, Orihel TC 2015 Ash & Orihel's atlas of human parasitology, 5th edn Chicago, IL: American Society for Clinical Pathology Press.

[51] Key FM, Posth C, Krause J, Herbig A, Bos KI 2017 Mining metagenomic data sets for ancient DNA: recommended protocols for authentication. Trends Genet. 33, 508–520. (10.1016/j.tig.2017.05.005) 28688671

[52] Briggs AW et al. 2007 Patterns of damage in genomic DNA sequences from a Neandertal. Proc. Natl Acad. Sci. USA 104, 14 616–14 621. (10.1073/pnas.0704665104) PMC1976210 17715061

[53] Dabney J, Meyer M, Pääbo S 2013 Ancient DNA damage. Cold Spring Harb. Perspect. Biol. 5, a012567 (10.1101/cshperspect.a012567) 23729639 PMC3685887

[54] Ginolhac A, Rasmussen M, Gilbert MTP, Willerslev E, Orlando L 2011 mapDamage: testing for damage patterns in ancient DNA sequences. Bioinformatics 27, 2153–2155. (10.1093/bioinformatics/btr347) 21659319

[55] Hofreiter M, Jaenicke V, Serre D, von Haeseler A, Pääbo S 2001 DNA sequences from multiple amplifications reveal artifacts induced by cytosine deamination in ancient DNA. Nucleic Acids Res. 29, 4793–4799. (10.1093/nar/29.23.4793) 11726688 PMC96698

[56] International Helminth Genomes Consortium. 2019 Comparative genomics of the major parasitic worms. Nat. Genet. 51, 163 (10.1038/s41588-018-0262-1) 30397333 PMC6349046

[57] Peltzer A, Jäger G, Herbig A, Seitz A, Kniep C, Krause J, Nieselt K 2016 EAGER: efficient ancient genome reconstruction. Genome Biol. 17, 60 (10.1186/s13059-016-0918-z) 27036623 PMC4815194

[58] Pasolli E et al. 2019 Extensive unexplored human microbiome diversity revealed by over 150,000 genomes from metagenomes spanning age, geography, and lifestyle. Cell 176, 649–662; e20. (10.1016/j.cell.2019.01.001) 30661755 PMC6349461

[59] Oh CS, Seo M, Chai JY, Lee SJ, Kim MJ, Park JB, Shin DH 2010 Amplification and sequencing of *Trichuris trichiura* ancient DNA extracted from archaeological sediments. J. Archaeol. Sci. 37, 1269–1273. (10.1016/j.jas.2009.12.029)

[60] Anderson TJC 2001 The dangers of using single locus markers in parasite epidemiology: *Ascaris* as a case study. Trends Parasitol. 17, 183–188. (10.1016/S1471-4922(00)01944-9) 11282508

[61] Dabney J et al. 2013 Complete mitochondrial genome sequence of a Middle Pleistocene cave bear reconstructed from ultrashort DNA fragments. Proc. Natl Acad. Sci. USA 110, 15 758–15 763. (10.1073/pnas.1314445110) PMC3785785 24019490

[62] Meyer M, Kircher M 2010 Illumina sequencing library preparation for highly multiplexed target capture and sequencing. Cold Spring Harb. Protoc. 2010, pdb.prot5448 (10.1101/pdb.prot5448) 20516186

[63] Li H, Durbin R 2009 Fast and accurate short read alignment with Burrows-Wheeler transform. Bioinformatics 25, 1754–1760. (10.1093/bioinformatics/btp324) 19451168 PMC2705234

[64] Oksanen J et al 2019 vegan: Community Ecology Package, package version 2.5-6. See https://CRAN.R-project.org/package=vegan.

[65] Wickham H 2016 Ggplot2: elegant graphics for data analysis. New York, NY: Springer.

[66] *Ascaris lumbricoides*—WormBase ParaSite [WWW Document], n.d. See https://parasite.wormbase.org/Ascaris_lumbricoides_prjeb4950/Info/Index (accessed 20 May 2019).

[67] Park J-K, Kim K-H, Kang S, Jeon HK, Kim J-H, Littlewood DTJ, Eom KS 2007 Characterization of the mitochondrial genome of *Diphyllobothrium latum* (Cestoda: Pseudophyllidea)—implications for the phylogeny of eucestodes. Parasitology 134, 749–759. (10.1017/S003118200600206X) 17214910

