# Supplementary material for: Estimating molecular preservation of the intestinal microbiome via metagenomic analyses of latrine sediments from two medieval cities
Source: Philos Trans R Soc Lond B Biol Sci. 2020 Oct 5;375(1812):20190576. doi: 10.1098/rstb.2019.0576 (PMC7702797; doi:10.1098/rstb.2019.0576)
Supplement: Supplementary figures [file rstb20190576supp1.docx]

**Supplementary Information**

For

*“Estimating molecular preservation of the intestinal microbiome via metagenomic analyses of latrine sediments from two medieval cities”*

Susanna Sabin,^1,2^ Hui-Yuan Yeh,^3^ Aleks Pluskowski,^4^ Christa Clamer,^5^ Piers D. Mitchell,^6^ Kirsten I. Bos^1^

1Max Planck Institute for the Science of Human History

2Center for Evolution and Medicine, Arizona State University, Tempe, AZ, USA

3 School of Humanities, Nanyang Technological University, 48 Nanyang Avenue, Singapore 639818.

4 Department of Archaeology, University of Reading, Whiteknights, Reading RG6 6AB, UK.

5 École Biblique de Jérusalem, P.O. Box 19053, IL9119001 Jerusalem.

6 Department of Archaeology, University of Cambridge, The Henry Wellcome Building, Fitzwilliam Street, Cambridge CB2 1QH, UK

Address correspondence to: [sabin@shh.mpg.de](mailto:sabin@shh.mpg.de) and bos@shh.mpg.de

**
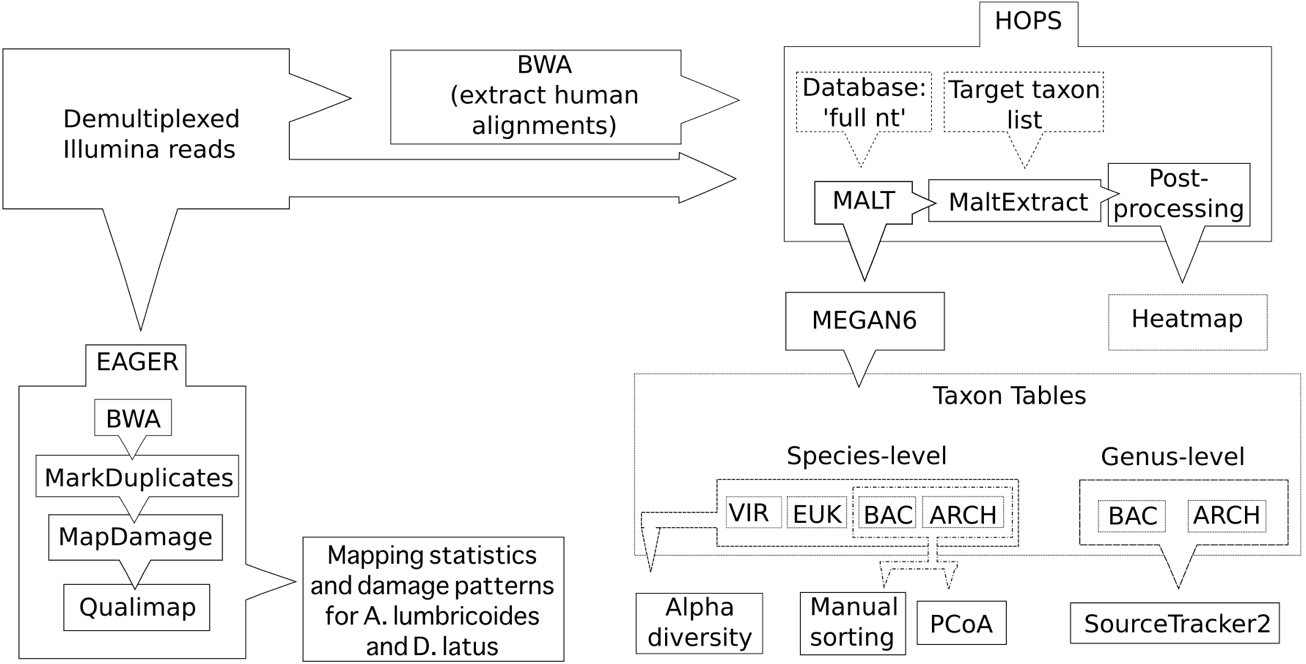
**

**Figure S1. Flowchart of computational analysis.** Starting from adapter-clipped, quality filtered reads combined between both sequencing runs (see METHODS) from Jerusalem and Riga samples, data was a) processed directly with HOPS and b) processed with HOPS after reads in the libraries aligning to the HG19 human reference genome were removed using BWA and Samtools. The (a) results were only used to produce taxon tables for alpha diversity.

**
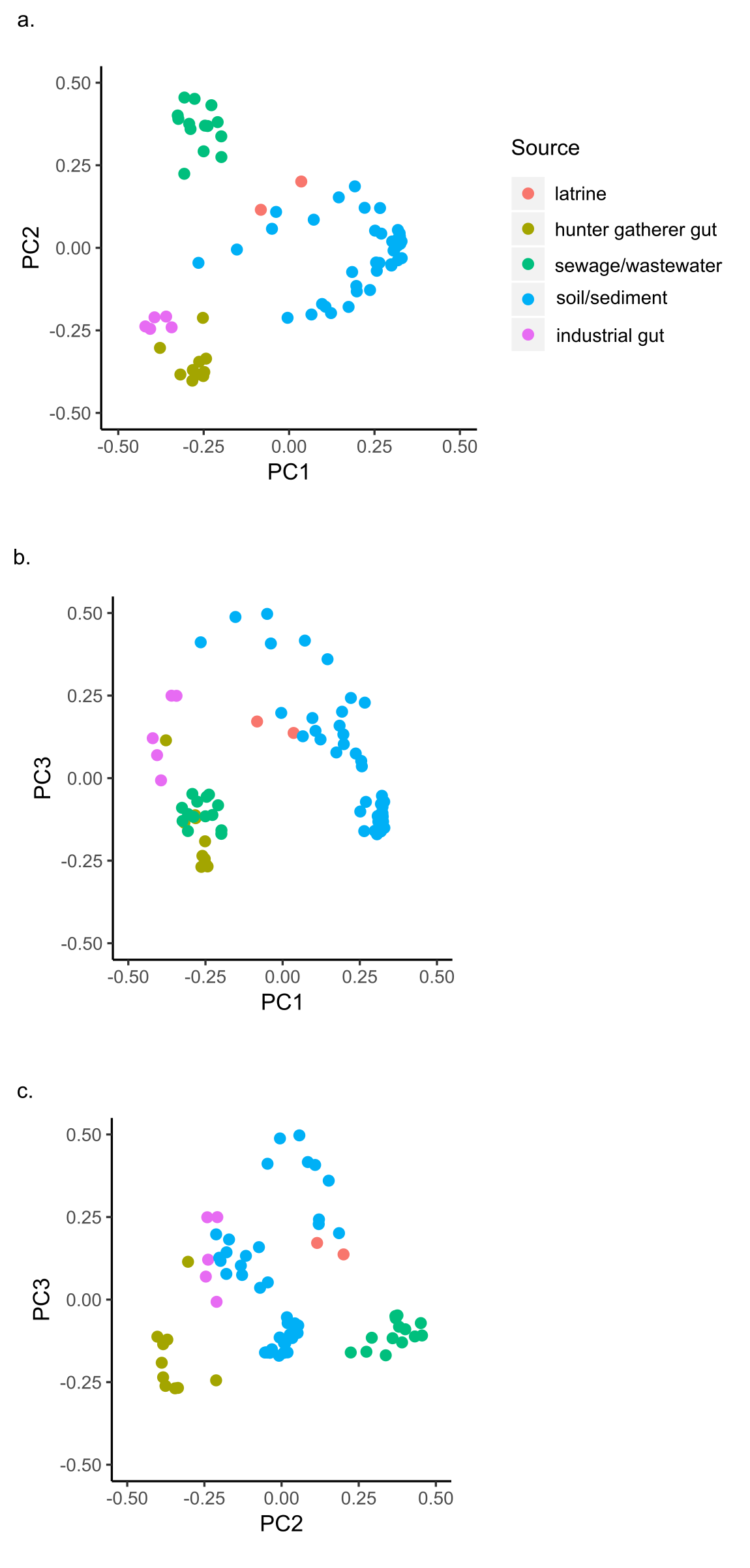
**

**Figure S2. PCoA of latrine and model source metagenomes based on Bray Curtis taxonomic distance.**

**
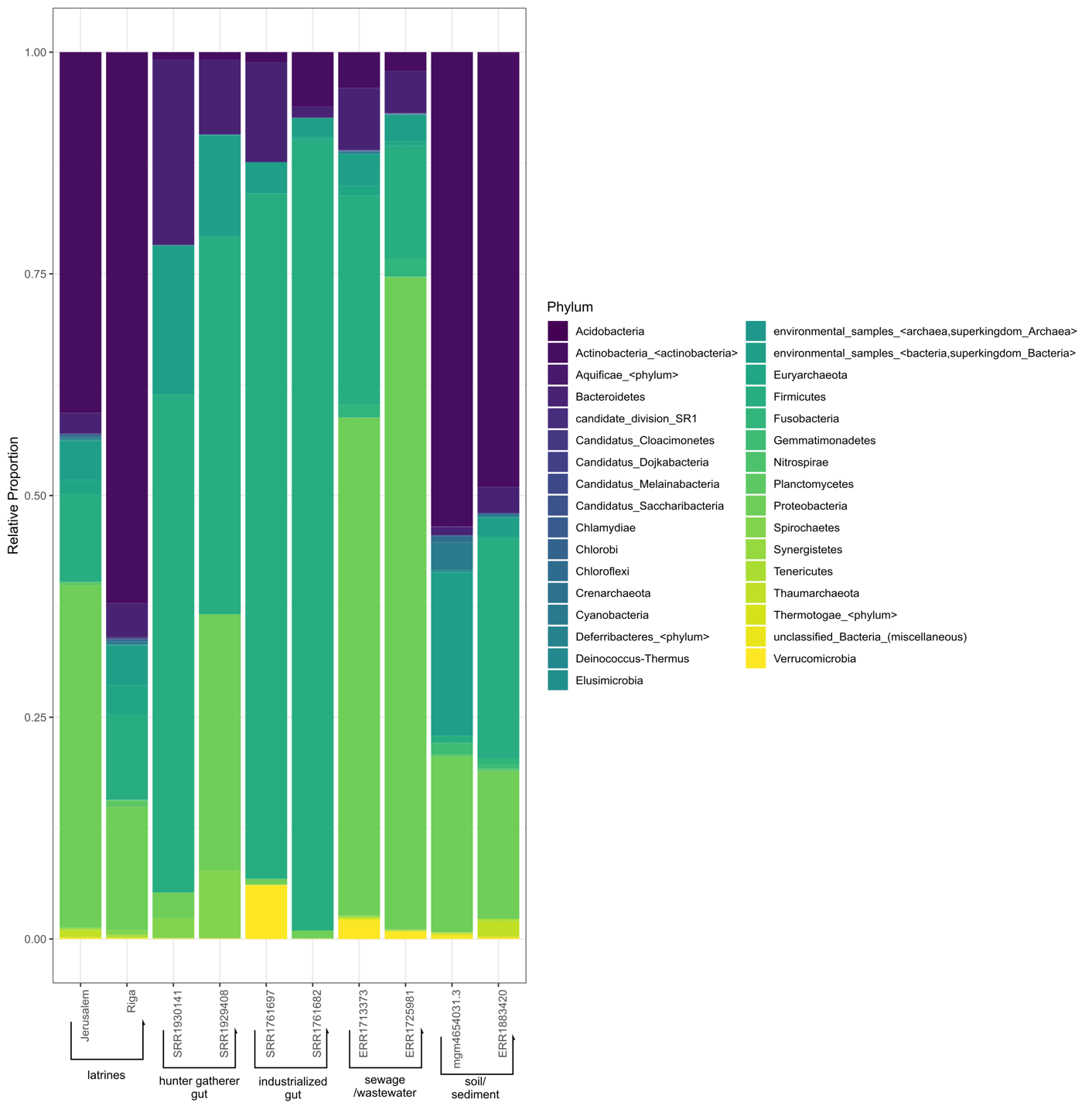
**

**Figure S3. Proportions of top 25 phyla in latrine samples and subset of source model metagenomes.**

**
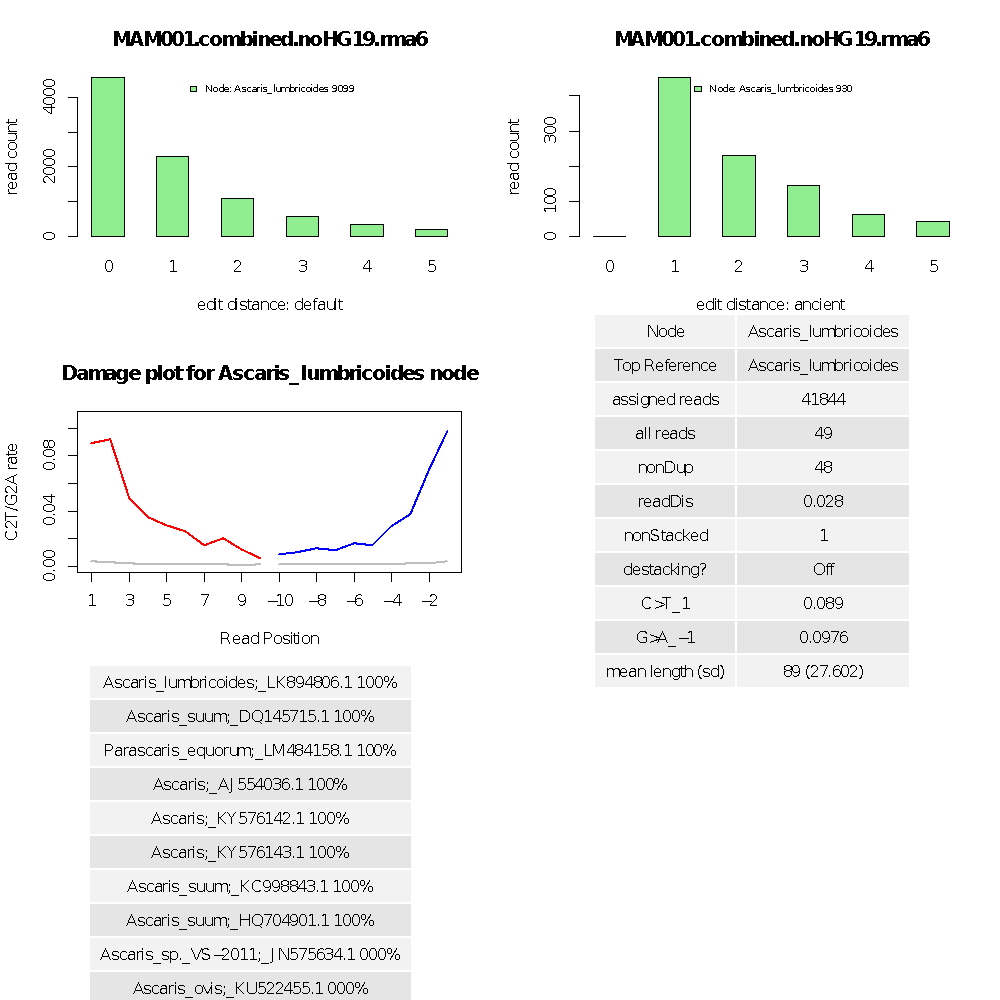
**

**Figure S4.** HOPS *Ascaris lumbricoides* report for Jerusalem combined library with human reads removed. The within-lab code for this sample is MAM001. The alignments share great similarity with other *Ascaris* sequences, but all are parasites that infect humans and domesticated animals. Edit distance: default refers to the number of mismatches to the node sequences per read among all aligned reads. Edit distance: ancient refers to the number of mismatches to the node sequences per read among aligned reads with at least one position of C>T damage.

**
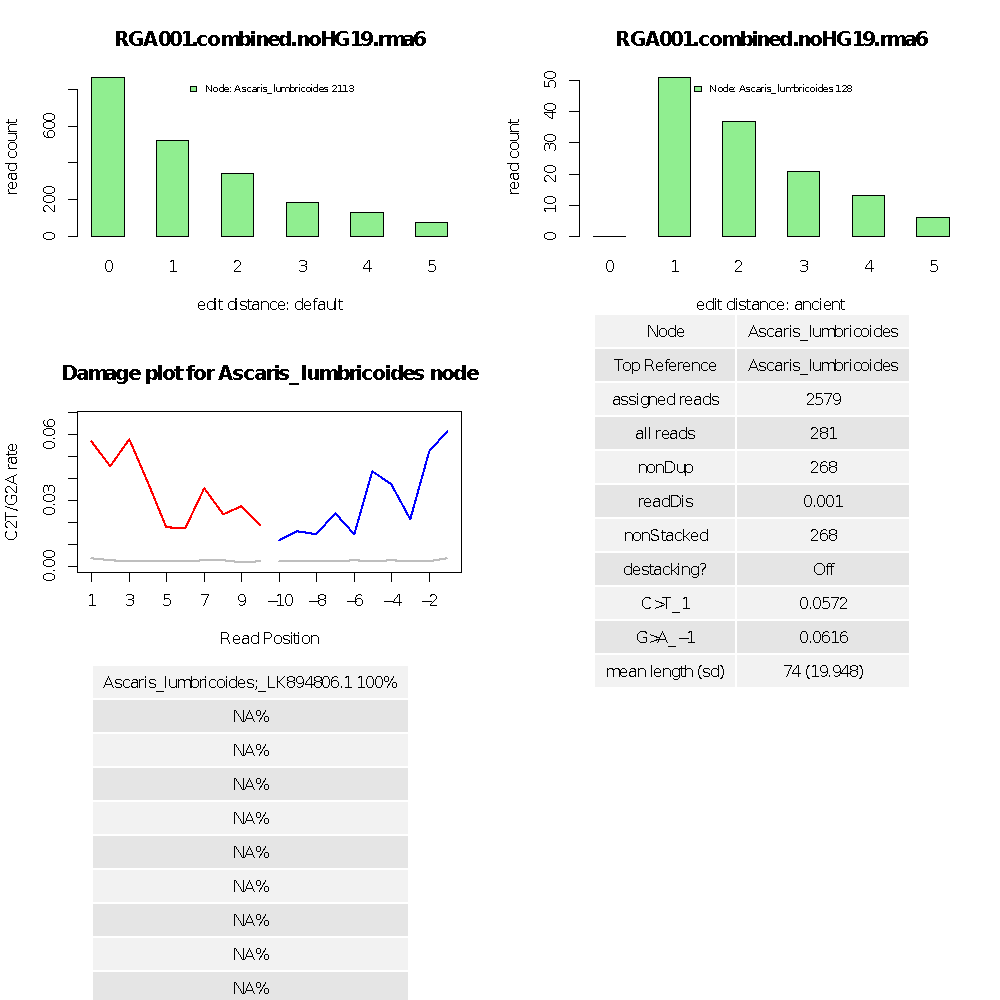
**

**Figure S5.** HOPS *Ascaris lumbricoides* report for Riga combined library with human reads removed. The within-lab code for this sample is RGA001. Edit distance: default refers to the number of mismatches to the node sequences per read among all aligned reads. Edit distance: ancient refers to the number of mismatches to the node sequences per read among aligned reads with at least one position of C>T damage.


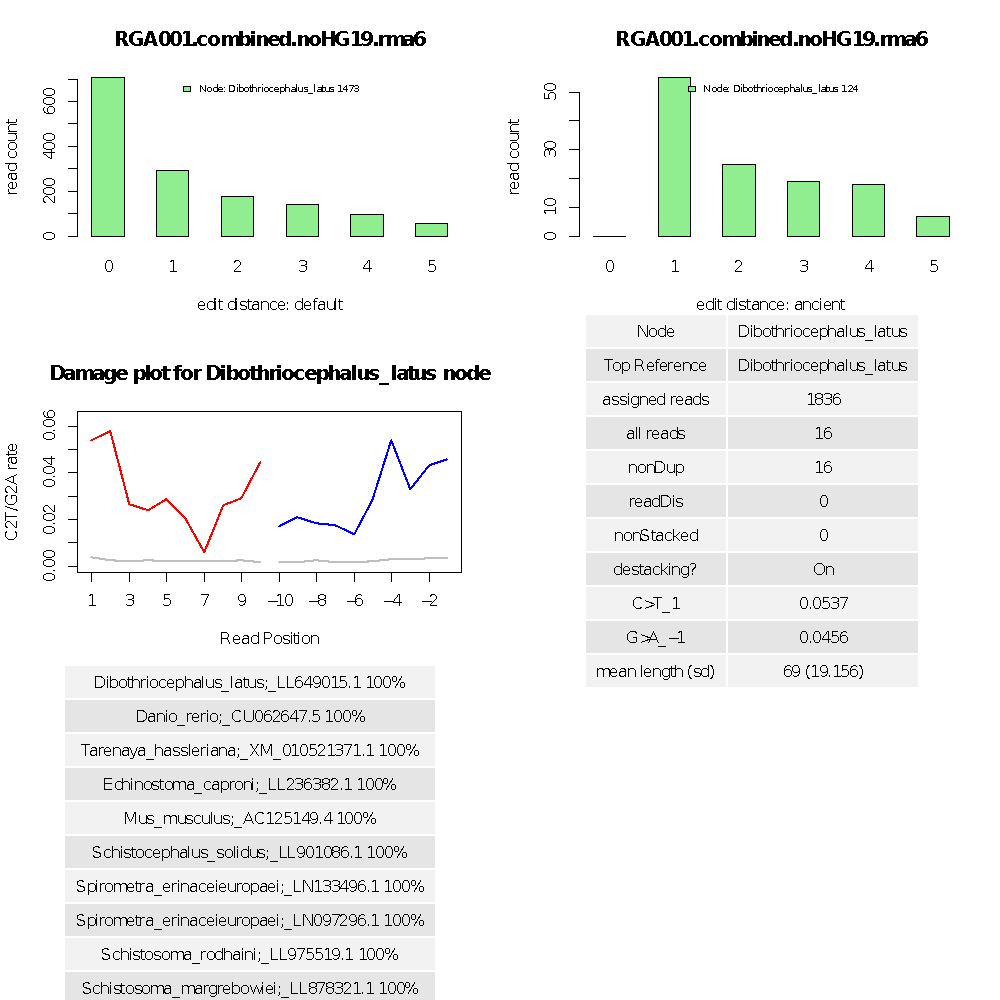


**Figure S6.** HOPS *Dibothriocephalus latus* report for Riga combined library with human reads removed. The sequences share identity with many other Eukaryotes, suggesting there could be misalignments in these MALT results. Independent of the paleoparasitological evidence for this parasite in the sediments, the true presence of *D. latus* would be questionable on metagenomic evidence alone. Edit distance: default refers to the number of mismatches to the node sequences per read among all aligned reads. Edit distance: ancient refers to the number of mismatches to the node sequences per read among aligned reads with at least one position of C>T damage.
